# Aloin Regulates Matrix Metabolism and Apoptosis in Human Nucleus Pulposus Cells via the TAK1/NF-*κ*B/NLRP3 Signaling Pathway

**DOI:** 10.1155/2022/5865011

**Published:** 2022-01-06

**Authors:** Taiqiu Chen, Pengfei Li, Jincheng Qiu, Wenjun Hu, Shaoguang Li, Huihong Shi, Xianjian Qiu, Dongsheng Huang, Wenjie Gao, Anjing Liang

**Affiliations:** Department of Orthopedics, Sun Yat-sen Memorial Hospital of Sun Yat-sen University, Guangzhou, Guangdong 510000, China

## Abstract

Intervertebral disc degeneration (IDD) is a degenerative disease that is characterized by decreased matrix synthesis and extra degradation, nucleus pulposus cells (NPCs) apoptosis, and infiltration of inflammatory factors. Aloin, a colored compound from aloe plants, has been shown to be effective against skeletal degenerative diseases, but it is unclear whether it is protective against IDD. Herein, we investigated the role of aloin in NPCs. In our study, the upregulation of proinflammatory factors, apoptosis, and unbalanced matrix metabolism were observed in degenerative NP tissues. We found that aloin had a curative effect on extracellular matrix metabolism and apoptosis in TNF-alpha- (TNF-*α*-) treated NPCs by inhibiting oxidative stress and the proinflammatory factor expression. Further investigation revealed that aloin treatment suppressed the TAK1/NF-*κ*B pathway. Moreover, the expression level of the NLPR3 inflammasome was downregulated after aloin treatment in TNF-*α*-treated NPCs. In summary, our results demonstrated that aloin treatment can reverse TNF-*α*-induced unbalanced matrix metabolism and apoptosis of NPCs via the TAK1/NF-*κ*B/NLRP3 axis. This study supports that aloin can be a promising therapeutic agent for IDD.

## 1. Introduction

Low back pain (LBP) is a universal symptom with a high incidence rate. Almost 80% of people have suffered from LBP in their lifetime, and the number continues to rise globally with the aging of the population [1, 2]. LBP is a serious problem for human health that reduces the quality of life, especially for young patients [3, 4]. It has been reported that the number of people with disabilities caused by LBP has increased in the past thirty years, especially in developing countries [5, 6].

Intervertebral disc degeneration (IDD) has been contemplated to be the primary cause of LBP. The intervertebral disc is made up of annulus fibrosus, nucleus pulposus, and endplates [7]. A variety of pathological changes are related to the etiology and progression of IDD, including unbalanced metabolism within the extracellular matrix (ECM), apoptosis of NPCs, and overexpression of inflammatory factors, including interleukin-1 beta and tumor necrosis factor-alpha [8, 9]. The ECM is secreted essentially by NPCs, and its destruction is characterized by inadequate anabolism and excessive catabolism, accompanied by the downregulation of collagen II and aggrecan and the upregulation of matrix metallopeptidases (MMPs) [10, 11]. Apoptosis of NPCs is also considered to be an important pathological change in IDD. Studies based on the nucleus pulposus tissue of IDD patients revealed an increased apoptosis rate and the overexpression of induced apoptosis factors in NPCs, including inflammatory factors and oxidative stress levels [12–14].

As an essential member of the TNF superfamily of ligands, TNF-*α* has been reported to be pivotally involved in the progression of IDD [15, 16]. It has been demonstrated that the concentration of TNF-*α* increases with the aggravation of IDD [17]. As the level of TNF-*α* increases, it can significantly enhance the expression of MMPs and ADAMTSs, and reduce the expression levels of aggrecan and collagen II, resulting in degradation of the ECM [18, 19]. Moreover, TNF-*α* has been considered to play a critical role in NPC apoptosis and can enhance the expression levels of multiple proinflammatory cytokines, such as cytochrome C oxidase subunit 2 (COX-2), and inducible nitric oxide synthase (iNOS), which amplify the inflammatory response and aggravate the IDD [20–22]. Thus, TNF-*α* was used to induce the degeneration of NPCs in our study.

The nuclear factor kappa B (NF-*κ*B) pathway was found to be linked to the occurrence and development of IDD, especially with inflammatory microenvironment irritation [23, 24]. With TNF-*α* stimulation, proteins upstream of the pathway, such as Ikk-alpha/beta and transforming growth factor-*β*-activated kinase 1 (TAK1), are stimulated to phosphorylate I*κ*B*α*. Phophorylated I*κ*B*α* then produces nuclear localization signals to activate and phosphorylate the p65 subunit of nuclear factor kappa B, which finally initiates the target gene transcription [25, 26]. Additionally, the NF-*κ*B pathway has been found to participate in the metabolism of the extracellular matrix, as well as apoptosis and autophagy of NPCs [27–29].

The NLR family pyrin domain containing 3 (NLRP3) inflammasome has been reported to be involved in the progression of various diseases [30]. As a target gene of phosphorylated p65, the NLRP3 inflammasome has been reported to be linked with IDD in previous studies [31–33]. In addition, the NLRP3 inflammasome might mediate the induction of inflammatory factors in NPCs, accelerating the progression of IDD.

Aloin, extracted from aloe plant species used in traditional medicinal medicine, has been shown to be effective both in vitro and in vivo against inflammation, skeletal degenerative disease, cancer, and cardiovascular diseases [34, 35]. Sun et al. found that aloin can diminish oxidative stress level and reduce the concentration of reactive oxygen in myocardial cells [36]. Another study found that aloin treatment can suppress the activation of the NF-*κ*B/NLRP3 signaling pathway [37]. Moreover, it has been reported that aloin ameliorates the development of osteoarthritis and shows a protective effect on anabolism and catabolism of ECM in chondrocytes through the PI3K/AKT/NF-*κ*B pathway [38]. However, the effect and underlying mechanism of aloin on NPCs remain unknown. Thus, based on the anti-inflammatory and bone-protective effects, we hypothesized that aloin may regulate both matrix metabolism and apoptosis in NPCs via the TAK1/NF-*κ*B/NLRP3 axis. Our study aims to provide additional evidence for aloin in therapy of IDD.

## 2. Material and Methods

### 2.1. Antibodies and Reagents

Antibodies against GAPDH, COL2A1, ACAN, ADAMTS4, ADAMTS5, MMP9, MMP13, TNF-*α*, NOX1, NOX2, iNOS, COX2, and NLRP3 were purchased from Abcam Inc. Catalase, SOD1, BAX, BCL2, Caspase3, Cleaved-caspase3, TAK1/NF-*κ*B signaling proteins (TAK1, phosphor-TAK1, P65, phosphor-P65, I*κ*B*α*, and phosphor-I*κ*B*α*), and IgG secondary antibodies were purchased from Cell Signaling Technology Inc. Antibody against GAPDH was from Proteintech Group Inc. Aloin reagent was from MedChemExpress, and human TNF-*α* was from R&D Systems. Abbreviations and descriptions were shown in Supplement Table 1.

### 2.2. Tissue Samples of Controls and Patients

Degenerative nucleus pulposus (NP) tissues were gained from patients who underwent discectomy surgery due to disc herniation, while the normal NP tissues were obtained from those who received a surgery due to trauma without disc degeneration. Tissues were fixed, decalcified, embedded and cut into sections for immunohistochemistry (IHC) analysis. Meanwhile, extra tissues were stored in -80°C for RNA and protein extraction by using high-throughput tissue grinder.

### 2.3. Human Nucleus Pulposus Cell Acquisition and Culture

In this study, the human nucleus pulposus cells (NPCs) were obtained from ScienCell. With the confluence of about 70%-90%, they were trypsinized and plated again. The NPCs were cultured in 6-well plate at a density of approximately 1.0 × 10^6^ cells/ml with about 8 ml human NPC medium (ScienCell) in 3-6 passages, and they were given the indicated treatment at 12 h after plating for subsequent experiments.

### 2.4. Immunohistochemistry

Paraformaldehyde was used to fix the tissues, and then, they were decalcified, dehydrated, and embedded. After being treated with 0.1% Triton X-100 solution for 15 minutes, the tissue sections were incubated with 3% peroxidase for 20 minutes and washed 3 times with PBS. As the blocking reagent, bovine serum albumin (5%, Sigma-Aldrich) was available at 37°C for 30 minutes. As follows, the Histostain Plus kit was used for IHC analysis. Finally, an Olympus BX63 microscope (Olympus, Tokyo, Japan) was used for photographing at magnifications of 200 and 400.

### 2.5. Real-Time qPCR

The total RNA was extracted from tissues and cells by using RNA-iso Plus reagent. After converting to cDNA, qPCR was performed, and the expression levels of mRNAs in different groups were calculated and analyzed referring to the expression level of *GAPDH* gene. All primers' sequences used for our research were included in Supplement Table 2.

### 2.6. Western Blot

Firstly, we extracted the total proteins from cells and tissues. Subsequently, samples were subjected in SDS-PAGE with equal amounts and transferred to NC transfer membranes. Next, the membranes were blocked and incubated with the designated antibodies at 4°C overnight. After being washed 3 times with PBS, they were incubated with the secondary antibodies for 1 h and then were visualized. The bands were determined and quantified using the ImageJ software.

### 2.7. Cell Viability Assay

NPCs were digested, centrifugated, and plated in 96-well plates at the density of 1.0 × 10^4^ cells/ml. After being cultured for 12 h, aloin at different concentrations was added for 24 and 48 h, respectively. NPCs were washed with PBS at the indicated time, and 10% CCK-8 reagent was added in each well. Finally, we measured the absorbance by using the microplate reader at 450 nm.

### 2.8. Immunofluorescence

NPCs were seeded on cover glasses in 24-well plates for 24 h and then were under different treatment for 48 h. Then, NPCs were washed, fixed, permeabilized, and blocked. Next, the cover glasses were incubated with antibody at 4°C overnight. After that, secondary antibodies (1 : 100) were used for incubation, and then, they were labelled with DAPI for 5 min. At last, an Olympus BX63 microscope was used for photographing.

### 2.9. Tunel Cell Apoptosis Detection

NPCs were seeded on cover glasses in 24-well plates for 24 h and then were under different treatments for 48 h. At the target confluence, NPCs were washed and fixed. Secondly, they were washed and treated with 0.1% Triton X-100 solution. Tunel working solution (Beyotime, China) was added, and images were photographed using an Olympus BX63 microscope.

### 2.10. Flow Cytometer

NPCs were cultured in the 6-well plates and collected after different treatments. Annexin V/PI apoptosis detection kit (Elabscience, China) was used to detect apoptotic rate. And then, binding buffer was added, and flow cytometry system was used for analyzing immediately.

### 2.11. Statistical Analysis

*t*-test for two comparisons or one-way ANOVA for multiple comparisons was used. All quantitative data were presented as the mean ± standard deviation. All analyses were conducted by using the GraphPad Software 8.0 (San Diego, CA, USA). Statistically significance was considered when *P* < 0.05.

### 2.12. Ethics Statement

Our research was approved by the Institutional Research Ethical Committee of Sun Yat-sen University (Guangzhou, China), and the written informed consent was obtained from all subjects participating in the study.

## 3. Results

### 3.1. Dysregulated ECM Metabolism, Increased Apoptosis, and Inflammatory Infiltration in Degenerated NP Tissue

**Figure 1:**
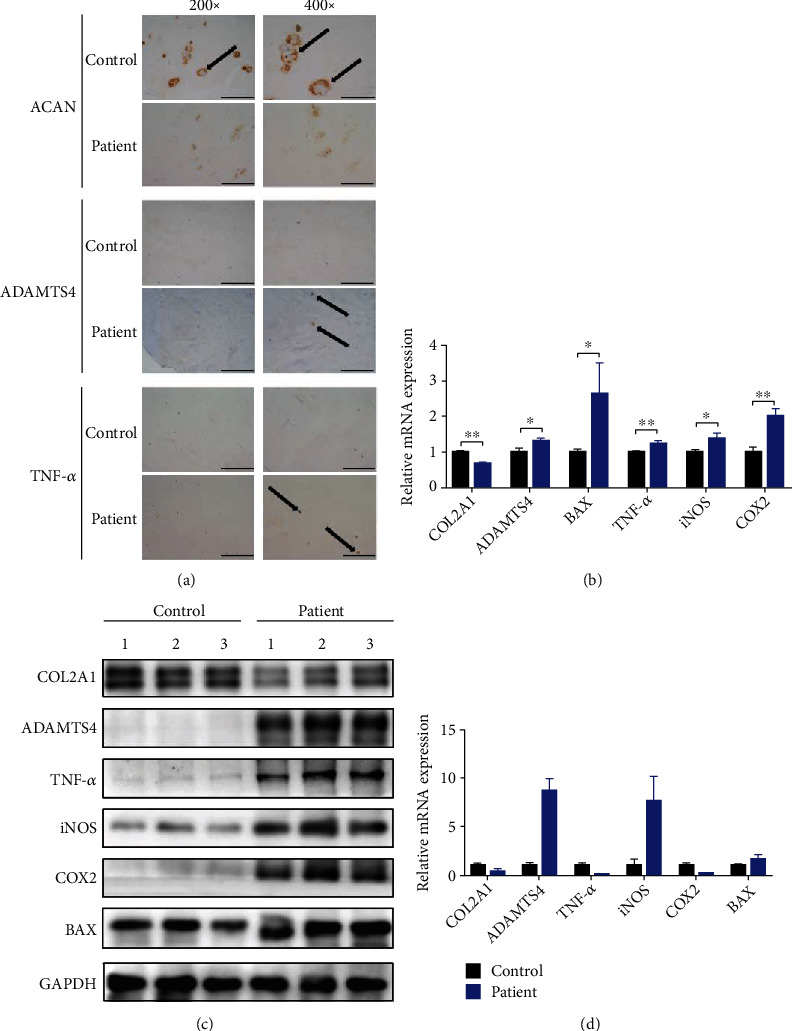
Dysregulated ECM metabolism, increased apoptosis, and inflammatory infiltration in degenerated NP tissue. (a) Immunohistochemical staining assay of ACAN, ADAMTS4, and TNF-*α* in normal and degenerated human NP tissues. (b) The mRNA expression levels of *COL2A1*, *ADAMTS4*, *BAX*, *TNF-α*, *iNOS*, and *COX2* were detected by qPCR in normal and degenerated human NP tissues. (c, d) Protein expression levels of COL2A1, ADAMTS4, BAX, TNF-*α*, iNOS, and COX2 were detected by western blotting in normal and degenerated human NP tissues. The densities of the protein bands were assessed and quantified using the ImageJ software. The left images (magnification: ×200, scale bar: 100 *μ*m) and the right images (magnification: ×400, scale bar: 50 *μ*m) are shown. ^∗^*P* < 0.05, ^∗∗^*P* < 0.01.

To determine the pathogenesis of IDD, immunohistochemistry was used to evaluate the expressions of ECM metabolism markers and inflammatory factors in different human NP tissues. We found that the expression of ACAN was significantly reduced, and the expressions of ADAMTS4 and TNF-*α* were upregulated in the patient group (Figure 1(a)). Protein and mRNA were then extracted from normal and degenerated human NP tissues and assessed. As shown in Figures 1(b)–1(d), the expression of *COL2A1* was suppressed, and the matrix-degrading enzyme *ADAMTS4* was increased in the patient group. Furthermore, the expression levels of *TNF-α*, the apoptotic gene *BAX*, and the proinflammatory factors *iNOS* and *COX2* were upregulated in degenerative tissues compared to normal tissues (Figures 1(b)–1(d)).

### 3.2. Aloin Treatment Enhances the Anabolism and Suppresses the Catabolism in TNF-*α*-Treated NPCs

**Figure 2:**
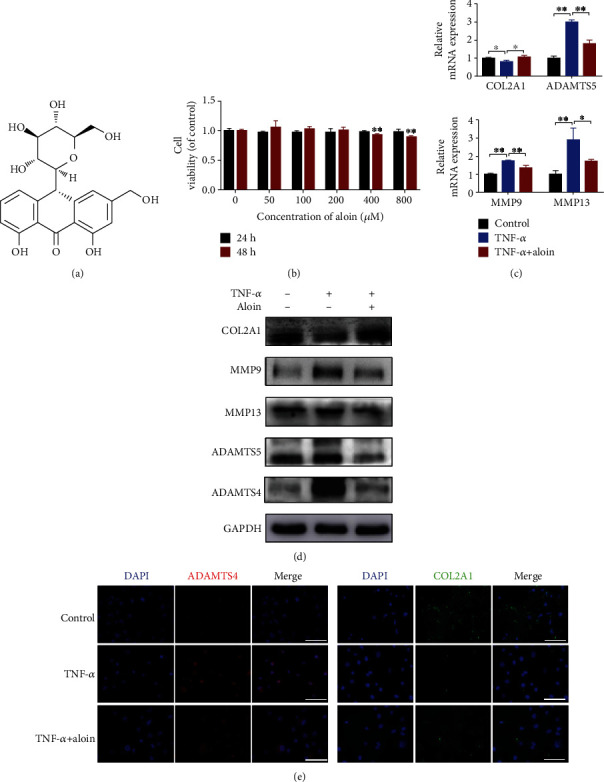
Aloin treatment enhances the anabolism and suppresses the catabolism in TNF-*α*-treated NPCs. (a) The chemical structure of aloin is shown (quoted from http://www.medchemexpress.cn/.) (b) Detection of aloin's cytotoxic effect on NPCs at various concentrations at 24 and 48 h posttreatment using a CCK-8 assay (0, 50, 100, 200, 400, and 800 *μ*M). (c) The mRNA expression levels of *COL2A1*, *ADAMTS5*, *MMP9*, and *MMP13* in the different groups were detected by qPCR. (d) Protein expression of COL2A1, ADAMTS5, MMP9, and MMP13 was detected by western blotting in the different groups. (e) Both ADAMTS4 and COL2A1 were detected by immunofluorescence staining. DAPI was used to stain for the nuclei. Images (magnification: ×400, scale bar: 50 *μ*m). In this (c–e), aloin was used at the concentration of 200 *μ*M, while TNF-*α* was at the concentration of 10 ng/ml. ^∗^*P* < 0.05, ^∗∗^*P* < 0.01.

The chemical structure of aloin is shown in Figure 2(a) (quoted from http://www.medchemexpress.cn/.). First, various concentrations of aloin were added to the medium (0, 50, 100, 200, 400, and 800 *μ*M), and we used the CCK-8 test to assess whether the cytotoxic effect of aloin on NPCs was exerted. As shown in Figure 2(b), we found that aloin had no significant cytotoxicity on the NPCs at concentrations up to 200 *μ*M; however, aloin reduced cell viability at concentrations of 400 *μ*M (*P* < 0.05) within 48 h. Next, we evaluated the expression levels of COL2A1, MMPs, and ADAMTSs by using qPCR and western blot, and the results showed that aloin treatment promoted ECM synthesis and significantly inhibited ECM degradation in TNF-*α*-treated NPCs (Figures 2(c) and 2(d)). As expected, the immunofluorescence analysis also showed that aloin treatment reversed the changes in ADAMTS4 and COL2A1 induced by TNF-*α* (Figure 2(e)).

### 3.3. Aloin Treatment Reduced the Apoptosis Rate in TNF-*α*-Treated NPCs

**Figure 3:**
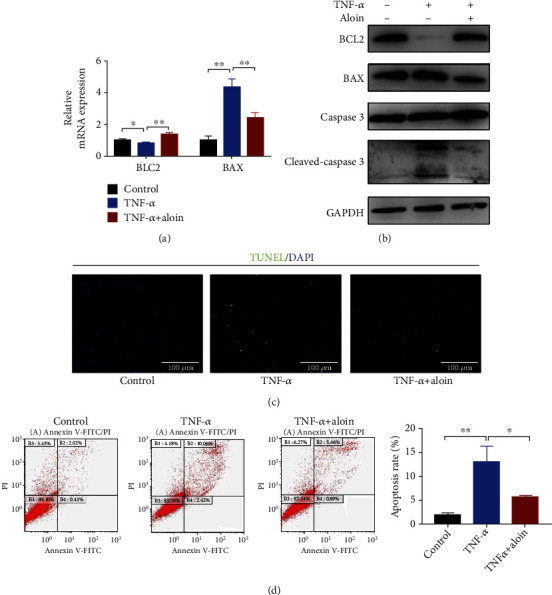
Aloin treatment reduced the apoptosis rate in TNF-*α*-treated NPCs. (a) The mRNA expression levels of *BCL2* and *BAX* were detected by qPCR in the different groups. (b) Protein expression levels of BCL2, BAX, Caspase3, and Cleaved-caspase3 were detected by western blotting in the different groups. (c) TUNEL immunofluorescence staining in the different groups. (d) Flow cytometry and apoptosis rate analysis in the different groups (*n* = 3). Images (magnification: ×200, scale bar: 100 *μ*m). In this figure, aloin was used at the concentration of 200 *μ*M, while TNF-*α* was at the concentration of 10 ng/ml. ^∗^*P* < 0.05, ^∗∗^*P* < 0.01.

Subsequently, apoptosis was detected in NPCs. The qPCR results showed that aloin treatment (200 *μ*M) in TNF-*α*-treated NPCs upregulated *BCL2* and downregulated *BAX* (Figure 3(a)). The western blot results also showed that the apoptotic markers BAX and Cleaved-caspase3 were downregulated, and the antiapoptotic marker BCL2 was upregulated with aloin treatment (Figure 3(b)). Next, to assess the apoptosis rate, TUNEL immunofluorescence staining and flow cytometry were used. As shown in Figures 3(c) and 3(d), we found a significantly higher apoptosis rate in TNF-*α*-treated NPCs, whereas it was inhibited with aloin treatment at a concentration of 200 *μ*M (Figures 3(c) and 3(d)). Our results indicated that aloin treatment can reduce the apoptosis rate in TNF-*α*-induced NPCs.

### 3.4. Aloin Treatment Ameliorates the Levels of Oxidative Stress and Proinflammatory Factors

**Figure 4:**
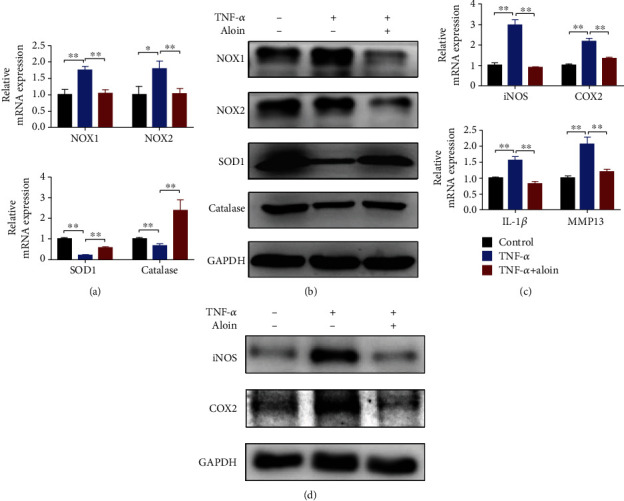
Aloin treatment ameliorates the levels of oxidative stress and proinflammatory factors. (a) The mRNA expression levels of *NOX1*, *NOX2*, *SOD1*, and *Catalase* were detected by qPCR. (b) Protein expression levels of NOX1, NOX2, SOD1, and Catalase were detected by western blotting. (c) The mRNA expression levels of *iNOS*, *COX2*, *IL-β*, and *IL-6* were detected by qPCR. (d) Protein expression levels of iNOS and COX2 were detected by western blotting. In this figure, aloin was used at the concentration of 200 *μ*M, while TNF-*α* was at the concentration of 10 ng/ml. ^∗^*P* < 0.05, ^∗∗^*P* < 0.01.

Then, we detected the effects of aloin treatment on proinflammatory factors and oxidative stress levels. The results indicated that markers of oxidation indices (*NOX1* and *NOX2*) were downregulated, and antioxidant enzymes (*SOD1* and *Catalase*) were upregulated with aloin treatment (200 *μ*M) (Figures 4(a) and 4(b)). In addition, the inhibition of proinflammatory factors (*iNOS*, *COX2*, *IL-1β*, and *IL-6*) was observed with aloin treatment by using qPCR (Figure 4(c)). The protein levels of iNOS and COX2 were downregulated in the aloin-treated group, as shown by using western blot analysis (Figure 4(d)). These data suggested that aloin can inhibit the levels of oxidative stress and the production of proinflammatory factors in TNF-*α*-induced NPCs.

### 3.5. Aloin Suppresses the Activation of the TAK1/NF-*κ*B Pathway

**Figure 5:**
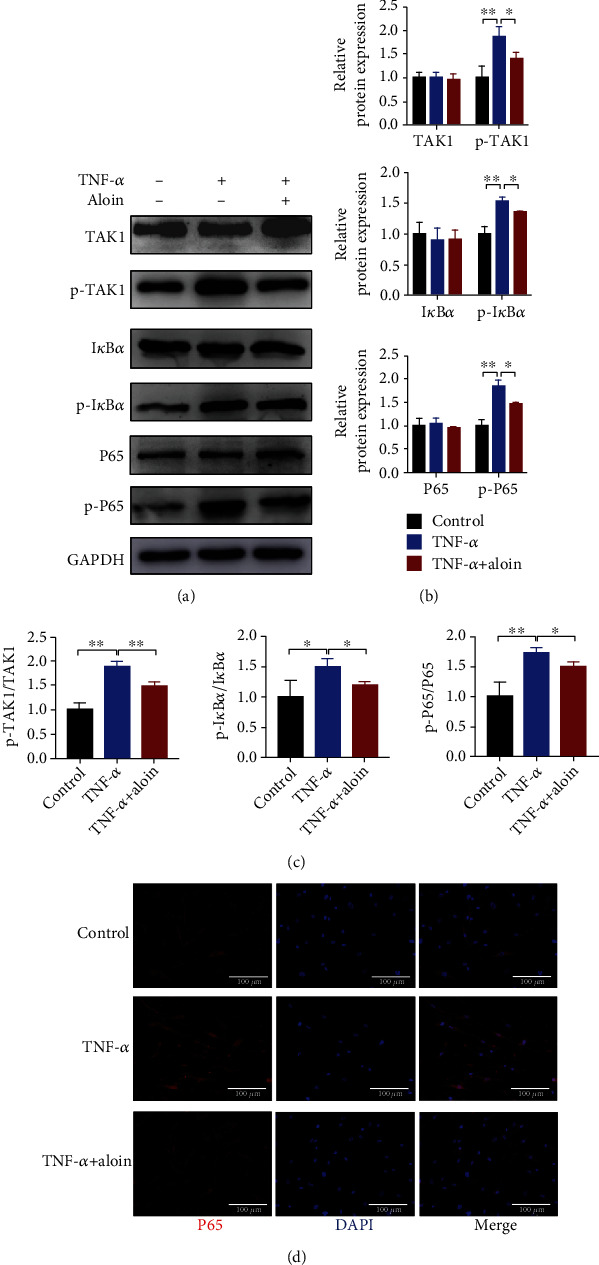
Aloin suppresses the activation of the TAK1/NF-*κ*B pathway. (a) Protein expression levels of the TAK1/NF-*κ*B signaling pathway (TAK1, p-TAK1, I*κ*B*α*, p-I*κ*B*α*, P65, p-P65) were detected by western blotting in different groups. (b) The protein bands were quantified using the ImageJ software. (c) The expression ratios of p-TAK1/TAK1, p-I*κ*B*α*/I*κ*B*α*, and p-P65/P65 in the different groups. (d) Localization of P65 was assessed via immunofluorescence. Images (magnification: ×200, scale bar: 100 *μ*m). In this figure, aloin was used at the concentration of 200 *μ*M, while TNF-*α* was at the concentration of 10 ng/ml. ^∗^*P* < 0.05, ^∗∗^*P* < 0.01.

Subsequently to explore the underlying mechanism, we evaluated the activation of the TAK1/NF-*κ*B axis under TNF-*α* treatment. As shown in Figures 5(a)–5(c), higher ratios of p-TAK1/TAK1, p-I*κ*B*α*/I*κ*B*α*, and p-P65/P65 were observed in TNF-*α*-treated NPCs, but aloin (200 *μ*M) treatment significantly reversed these changes in the ratios of p-TAK1/TAK1, p-I*κ*B*α*/I*κ*B*α*, and p-P65/P65 (Figures 5(a)–5(c)). We further assessed the localization of P65 via immunofluorescence and found that the proportion of P65 translocated from the cytoplasm into the nucleus was upregulated under TNF-*α* treatment, whereas aloin treatment attenuated the translocation of P65 (Figure 5(d)). These results suggested that aloin treatment can suppress the activation of the TAK1/NF-*κ*B pathway.

### 3.6. Aloin Treatment Inhibits the Expression of the NLRP3 Inflammasome

**Figure 6:**
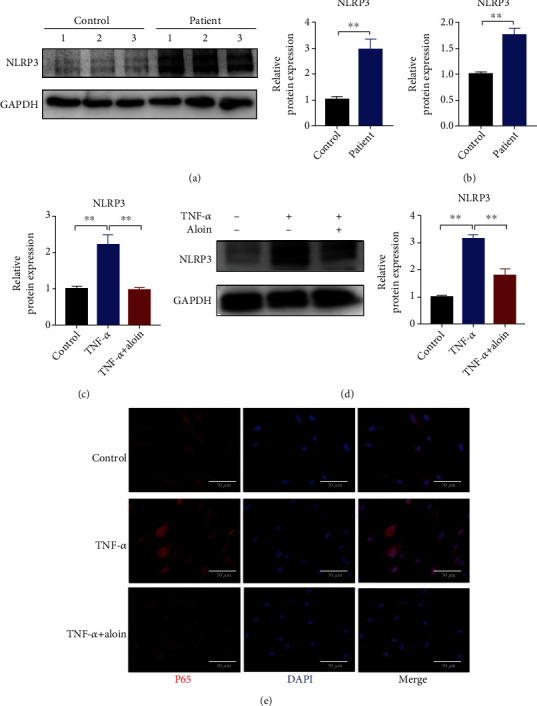
Aloin treatment inhibits the expression of the NLRP3 inflammasome. (a) The expression level of NLRP3 was detected by western blot in normal and degenerated human NP tissues. The protein bands were quantified using the ImageJ software. (b) The mRNA level of *NLRP3* was detected by qPCR in normal and degenerated human NP tissues. (c) The mRNA expression of *NLRP3* was detected by qPCR in the different groups. (d) The expression of NLRP3 was detected at the protein level by western blotting (left panel). The protein bands were quantified by the ImageJ software (right panel). (e) NLRP3 detection by using immunofluorescence. Images (magnification: ×400, scale bar: 50 *μ*m). In this figure, aloin was used at the concentration of 200 *μ*M, while TNF-*α* was at the concentration of 10 ng/ml. ^∗^*P* < 0.05, ^∗∗^*P* < 0.01.

As reported previously, the NLRP3 inflammasome is involved in inflammation-related NPCs and the progression of IDD [32]. To further investigate whether aloin regulated NLRP3 inflammasome activity in NPCs in an inflammatory microenvironment, we assessed the level of the NLRP3 inflammasome and found that it was higher in degenerated disc tissue than in normal tissues at both the mRNA and protein levels (Figures 6(a) and 6(b)). We then observed the expression of the NLRP3 inflammasome was inhibited upon aloin treatment by qPCR and western blot in TNF-*α*-treated NPCs (Figures 6(c) and 6(d)). In addition, immunofluorescence also showed that aloin treatment downregulated the expression of *NLRP3* in TNF-*α*-treated NPCs. These results suggested that aloin treatment reduced the expression of *NLRP3*.

## 4. Discussion

IDD, a common degenerative disease, is also considered to be the main cause of the high incidence of LBP, which causes pain and even disability [3, 4, 39]. However, searching for effective treatment drugs for IDD is still a huge challenge. Aloin, extracted from aloe plants, has been shown to have anti-inflammatory, antioxidant, and bone protective activities, but its effects on IDD have not been confirmed [37, 38, 40]. In our study, we demonstrated that aloin treatment can reverse TNF-*α*-induced extracellular matrix metabolism disorder, apoptosis, oxidative stress, and the production of proinflammatory factors in NPCs through the TAK1/NF-*κ*B/NLRP3 pathway. These results indicated that aloin may be a promising therapeutic method for IDD.

Aloin has been shown to have important protective effects on the skeletal system, including ameliorating the progression of osteoarthritis, promoting the expression levels of osteoblast differentiation genes (*BMP2* and *RUNX2*) in a dose-dependent manner, and suppressing osteoclastogenesis and bone resorption in previous studies [38, 41–43]. To assess the potential protective effect on NPCs, we first determined nontoxic concentrations of aloin in NPCs. We further demonstrated that aloin can reverse extracellular matrix metabolism disorders, apoptosis, oxidative stress, and the production of proinflammatory factors in TNF-*α*-treated NPCs in vitro. Similarly, a previous study showed that aloin treatment significantly inhibited both inadequate anabolism and excessive catabolism of the extracellular matrix in chondrocytes [38], while another study found that aloin effectively increased the activity of antioxidant enzymes and inhibited the levels of reactive oxygen species [44].

Previous studies have shown that TNF-*α* plays a conclusive role in the occurrence and development of IDD; thus, inhibiting inflammation effectively can ameliorate the progression of IDD [15–17, 45–47]. It has been demonstrated that aloin is effective in reducing the production of inflammatory and proinflammatory factors [37, 38]. A previous study reported that aloin treatment significantly decreased the expression of iNOS and TNF-*α* [48], while other studies found that aloin exerted anti-inflammatory activity and reduced the release of inflammatory cytokines [49, 50]. Correspondingly, we demonstrated the protective effects of aloin on TNF-*α*-treated NPCs.

In our study, we demonstrated that aloin treatment suppressed the activation of the TAK1/NF-*κ*B pathway and inhibited the level of NLRP3 inflammasome. As previous studies had reported, the TAK1/NF-*κ*B/NLRP3 pathway played an important role in TNF-*α*-induced inflammation in NPCs [51, 52]. Our study confirmed that the importance of this inflammation-related signaling pathway in the progression of IDD. Similarly, it had been reported that aloin treatment inhibited the activation of NF-*κ*B signaling in lipopolysaccharide-induced acute lung injury, IL-1 beta-stimulated chondrocytes, and D-galactose-induced cognitive impairment and inflammation [37, 38, 44]. Our results provided more evidences on the powerful anti-inflammatory activity of aloin on NPCs.

LBP, which is associated with IDD, is widespread and has a high incidence. However, existing treatments do not counteract the IDD process. In our study, a new potential treatment was proposed. Aloin, with obvious effects including regulating ECM metabolism, inhibiting apoptosis, and anti-inflammatory and antioxidant activity in NPCs, may be a therapeutic agent for IDD. However, further study is essential to elucidate the effect of aloin on IDD in vivo.

## 5. Conclusion

We assessed the protective effects of aloin on NPCs and demonstrated the underlying mechanism. In our study, we discovered that aloin promoted extracellular matrix homeostasis, inhibited apoptosis, and reduced the production of proinflammatory factors and the levels of oxidative stress in TNF-*α*-induced NPCs. In mechanistically, we found that aloin treatment suppressed the activation of the TAK1/NF-*κ*B/NLRP3 pathway. Our research indicates that aloin may be a therapeutic agent for IDD in the future.

## Data Availability

The data used to support the findings of this study are available from the corresponding author upon request.

## Supplementary Materials

Supplementary Materials Supplement Table 1: abbreviations and descriptions. Supplement Table 2: primers' sequences for RT-qPCR.
