# Supplementary material for: Aloin Regulates Matrix Metabolism and Apoptosis in Human Nucleus Pulposus Cells via the TAK1/NF-κB/NLRP3 Signaling Pathway
Source: Stem Cells Int. 2022 Jan 6;2022:5865011. doi: 10.1155/2022/5865011 (PMC8758297; doi:10.1155/2022/5865011)
Supplement: Supplementary Materials — Supplement Table 1: abbreviations and descriptions. Supplement Table 2: primers' sequences for RT-qPCR. [file 5865011.f1.docx]

Supplement Table 1. Abbreviations and descriptions.

| Abbreviations | | Descriptions |
| --- | --- | --- |
| COL2A1  ACAN  ADAMTS4  ADAMTS5  MMP9  MMP13  TNF-α  NOX1  NOX2  COX2  NLRP3  SOD1  BAX  IL-1β  IL-6  TAK1  GAPDH | Collagen type II  Aggrecan  A disintegrin-like and metalloprotease with thrombospondin type-1 motifs 4  A disintegrin-like and metalloprotease with thrombospondin type-1 motifs 5  Matrix metalloproteinase 9  Matrix metalloproteinase 13  Tumor necrosis factor-alpha  NADPH oxidase1  NADPH oxidase2  cytochrome c oxidase subunit II  NLR family pyrin domain containing 3  Superoxide dismutase 1  BCL2 associated X apoptosis regulator  Interleukin-1 beta  Interleukin-6  Transforming growth factor-β-activated kinase 1  Glyceraldehyde-3-phosphate dehydrogenase | |

Supplement Table 2. Primers’ sequences for RT-qPCR.

| Gene primer names | Primer sequence(5’-3’) |
| --- | --- |
| GAPDH-F  GAPDH-R  COL2A1-F  COL2A1-R  ADAMTS4-F  ADAMTS4-R  BAX-F  BAX-R  TNF-α-F  TNF-α-R  iNOS-F  iNOS-R  COX2-F  COX2-R  ADAMTS5-F  ADAMTS5-R  MMP9-F  MMP9-R  MMP13-F  MMP13-R  BCL2-F  BCL2-R  NOX1-F  NOX1-R  NOX2-F  NOX2-R  SOD1-F  SOD1-R  Catalase-F  Catalase-R  IL-1β-F  IL-1β-R  IL-6-F  IL-6-R  NLRP3-F  NLRP3-R | AGAAAAACCTGCCAAATATGATGAC  TGGGTGTCGCTGTTGAAGTC  GGCAATAGCAGGTTCACGTACA  GAACATCGACCAACTCTACTCCG  GAGGAGGAGATCGTGTTTCCA  CCAGCTCTAGTAGCAGCGTC  CCCGAGAGGTCTTTTTCCGAG  CCAGCCCATGATGGTTCTGAT  CCTCTCTCTAATCAGCCCTCTG  GAGGACCTGGGAGTAGATGAG  TTCAGTATCACAACCTCAGCAAG  TGGACCTGCAAGTTAAAATCCC  GCACCCCGACATAGAGAGC  CTGCGGAGTGCAGTGTTCT  GAACATCGACCAACTCTACTCCG  CAATGCCCACCGAACCATCT  TGTACCGCTATGGTTACACTCG  GGCAGGGACAGTTGCTTCT  ACTGAGAGGCTCCGAGAAATG  GAACCCCGCATCTTGGCTT  GGTGGGGTCATGTGTGTGG  CGGTTCAGGTACTCAGTCATCC  GCACACCTGTTTAACTTTGACTG  GGACTGGATGGGATTTAGCCA  AACGAATTGTACGTGGGCAGA  GAGGGTTTCCAGCAAACTGAG  GGTGGGCCAAAGGATGAAGAG  CCACAAGCCAAACGACTTCC  TGGGATCTCGTTGGAAATAACAC  TCAGGACGTAGGCTCCAGAAG  ATGATGGCTTATTACAGTGGCAA  GTCGGAGATTCGTAGCTGGA  ACTCACCTCTTCAGAACGAATTG  CCATCTTTGGAAGGTTCAGGTTG  GATCTTCGCTGCGATCAACAG  CGTGCATTATCTGAACCCCAC |
